# Liver injury associated with the severity of COVID-19: A meta-analysis

**DOI:** 10.3389/fpubh.2023.1003352

**Published:** 2023-02-02

**Authors:** Ruiqi Yang, Jihua Feng, Huan Wan, Xiaona Zeng, Pan Ji, Jianfeng Zhang

**Affiliations:** ^1^Department of Emergency Medicine, The Second Affiliated Hospital of Guangxi Medical University, Nanning, China; ^2^Department of General Practice, The Second Affiliated Hospital of Guangxi Medical University, Nanning, China

**Keywords:** COVID-19, coronavirus, liver injury, liver chemistry, meta-analysis

## Abstract

**Background:**

The current 2019 novel coronavirus disease (COVID-19) pandemic is a major threat to global health. It is currently uncertain whether and how liver injury affects the severity of COVID-19. Therefore, we conducted a meta-analysis to determine the association between liver injury and the severity of COVID-19.

**Methods:**

A systematic search of the PubMed, Embase, and Cochrane Library databases from inception to August 12, 2022, was performed to analyse the reported liver chemistry data for patients diagnosed with COVID-19. The pooled odds ratio (OR), weighted mean difference (WMD) and 95% confidence interval (95% CI) were assessed using a random-effects model. Furthermore, publication bias and sensitivity were analyzed.

**Results:**

Forty-six studies with 28,663 patients were included. The pooled WMDs of alanine aminotransferase (WMD = 12.87 U/L, 95% CI: 10.52–15.23, *I*^2^ = 99.2%), aspartate aminotransferase (WMD = 13.98 U/L, 95% CI: 12.13–15.83, *I*^2^ = 98.2%), gamma-glutamyl transpeptidase (WMD = 20.67 U/L, 95% CI: 14.24–27.10, *I*^2^ = 98.8%), total bilirubin (WMD = 2.98 μmol/L, 95% CI: 1.98–3.99, *I*^2^ = 99.4%), and prothrombin time (WMD = 0.84 s, 95% CI: 0.46–1.23, *I*^2^ = 99.4%) were significantly higher and that of albumin was lower (WMD = −4.52 g/L, 95% CI: −6.28 to −2.75, *I*^2^ = 99.9%) in severe cases. Moreover, the pooled OR of mortality was higher in patients with liver injury (OR = 2.72, 95% CI: 1.18–6.27, *I*^2^ = 71.6%).

**Conclusions:**

Hepatocellular injury, liver metabolic, and synthetic function abnormality were observed in severe COVID-19. From a clinical perspective, liver injury has potential as a prognostic biomarker for screening severely affected patients at early disease stages.

**Systematic review registration:**

https://www.crd.york.ac.uk/prospero/, Identifier: CRD42022325206.

## 1. Introduction

The outbreak of severe acute respiratory syndrome coronavirus 2 (SARS-CoV-2) disease in Wuhan, China (1), in December 2019 has caused a large number of infections and spread across the world (2, 3). By the end of January 2020, the infection was termed coronavirus disease 2019 (COVID-19) by the World Health Organization (WHO). Up to August 12, 2022, more than 585 million cases and more than 6 million deaths have been reported globally (4).

While respiratory and systemic symptoms such as fever, fatigue, and myalgia have been reported to be the main manifestations of symptomatic COVID-19, the literature has also reported liver injury (5, 6). The prevalence of elevated serum levels of aspartate aminotransferase (AST), alanine aminotransferase (ALT), alkaline phosphatase (ALP), lactate dehydrogenase (LDH), and gamma-glutamyl transpeptidase (GGT) have been observed.

The liver is the body's largest independent organ and serves four main functions: metabolism and synthesis; storage; excretion; and detoxification of potential poisons. Tests for indicators of hepatocellular injury (ALT, AST, GGT, and ALP), liver metabolic function [total bilirubin (TBIL)], and liver synthetic function [serum albumin (ALB) and prothrombin time (PT)] should be performed to fully assess liver injury (7).

It is currently uncertain whether and how liver injury affects the severity and mortality of COVID-19. Recent studies suggest a significant prevalence of abnormal aminotransferase levels in patients with COVID-19, including elevated AST, ALT, and total bilirubin (TBIL) levels and decreased albumin (ALB) levels (8). Previous studies have also suggested that the most severely affected patients present with coagulopathy, such as a prolonged PT, an increased international normalized ratio (INR), and disseminated intravascular coagulation (DIC) (9). However, a meta-analysis related to coagulopathy, mortality and COVID-19 and a comprehensive description of the four major functions of liver injury are rare. Therefore, we conducted a meta-analysis to determine the association between liver injury and the severity and mortality of COVID-19. We aimed to compare the risk and clinical outcomes of coagulopathy, hepatocellular injury, and liver synthesis and metabolic impairment in adult patients with severe and non-severe COVID-19 and provide a potential prognostic biomarker for screening severe patients at early stages of the disease for clinical treatment.

## 2. Methods

### 2.1. Database resource and search strategy

We conducted a systematic search of the PubMed, Embase, and Cochrane Library databases from December 2019 to August 12, 2022, using the following combined text and MESH terms: [“hepatitis” or “Liver disease” or “acute liver injury” or “liver injury”] and [“COVID-19” or “SARS-CoV-2” or “coronavirus”] and [“Pneumonia” or “Pneumonitis” or “Pulmonary Inflammation”] (full search terms available in Supplementary Table 1). Only studies available online were included, and the search was restricted to articles related to adult human studies published in English. Furthermore, we manually searched the relevant articles according to the inclusion criteria. The guidelines of the Preferred Reporting Items for Systematic Reviews and Meta-Analyses (PRISMA) statement were used to report all studies identified using a predefined search protocol detailed in Appendix 1, and the present study was registered with the International Prospective Register of Systematic Review (10) (number CRD42022325206).

### 2.2. Study eligibility and outcomes

Studies were included in the meta-analysis according to the following criteria: (i) participants were adult humans, regardless of underlying chronic liver disease (CLD) or COVID-19 severity; (ii) reverse transcription-polymerase chain reaction (RT–PCR)-confirmed COVID-19 cases were reported (11); (iii) reported one of the following serum parameters: liver biochemical parameters (serum ALT, AST, GGT, TBIL, or ALB), or blood coagulation function [serum PT, activated partial thromboplastin time (APTT), or INR] and the mean (SD) or median (IQR) levels in severe and non-severe cases of COVID-19 (or ICU and non-ICU patients) were reported; (iv) quantitative or qualitative laboratory data were reported; and (v) the characteristics and demographic information of the patients along with the publication year, country, number of patients, age, and sex were available. The exclusion criteria were as follows: (i) any study with subjects < 18 years old; (ii) case reports or case series with fewer than 20 subjects; (iii) review articles, editorials, guidelines, letters, notes and abstracts only; and (iv) non-human studies.

Our primary outcomes included biochemical parameters of hepatocellular injury (mean serum ALT, AST, and GGT), liver synthetic function (mean serum ALB), coagulation function (PT, APTT, and INR) and liver excretory function (mean serum TBIL) between severe and non-severe cases. We also assessed the incidence of liver injury in severe and non-severe cases, as well as the difference in mortality between patients with and without liver injury. The secondary outcomes consisted of sex (male, female), year, sample size, country, age and baseline CLD.

We defined elevated serum ALT or AST levels as those >40 U/L and elevated serum GGT levels as those >50 U/L. We defined hyperbilirubinemia as a TBIL level >17 μmol/L and hypoalbuminemia as a serum ALB level < 30 g/L. Elevation serum PT, APTT, and INR values were defined as values >13.5 s, 40 s, and 1.3, respectively.

The severity of COVID-19 was divided into three groups: mild, severe and critical according to the diagnosis and treatment standard of COVID-19 issued by the National Health Committee (4, 11, 12). Mild cases may present with fever and respiratory symptoms, and pneumonia was revealed by imaging. Severe COVID-19 was defined when the patients met at least one of the following criteria: (a) breathing rate ≥30/min; (b) resting oxygen saturation ≤ 93%; and (c) an FiO_2_ ratio (ratio of the partial pressure of arterial oxygen to the fraction of inspired oxygen) < 300 mmHg; In the critical group, at least one of the following three diagnostic criteria should be met: (i) respiratory failure requiring mechanical oxygenation; (ii) shock; and (iii) the development of other organ failure that necessitated intensive care unit (ICU) care. We defined the above severe and critical groups as severe cases in our study.

### 2.3. Data extraction and quality assessment

The selected articles were independently screened by three reviewers to assess eligibility for inclusion. The following data were extracted from the included studies into an Excel database: first author's name, article publication date, study design, sample size, patient age and sex, and outcome indicators (median and range, mean and SD, or overall prevalence). A fourth reviewer resolved any cases of disagreement. When necessary, the corresponding author was contacted through email for further information or clarification.

The quality of the included studies was assessed based on the Newcastle–Ottawa Scale (NOS) guidelines (13). Studies scoring more than 5 points were considered high quality, and those scoring 0–4 points were considered low quality. Three reviewers assessed study quality and resolved any disagreements by discussion.

### 2.4. Statistical analysis

The outcome indicators combined in this meta-analysis mainly included binary data and continuous data. When meta-analysis is performed on continuous data, the statistical value of the union is “mean difference” (MD), that is, the mean difference between the two groups. To obtain MD, it is necessary to obtain the sample size, mean and standard deviation of the two sets of data respectively. In the process of data extraction from the literature, the required data may not be extracted directly, thus affecting further data consolidation. Therefore, we conducted some data processing to obtain sample size, mean and standard deviation based on the method in previous researches by Hozo et al. (14) and Wan et al. (15).

Then, We analyzed the mean serum ALT, AST, GGT, TBIL, ALB, PT, APTT, and INR between severe and non-severe cases as continuous variables using the weighted mean difference (WMD) and 95% confidence interval (95% CI). For categorical outcomes, including incidence of liver injury in severe and non-severe cases, and mortality between patients with and without liver injury, the pooled odds ratio (OR) and 95% CI were calculated.

We used a random-effects model and validated our results by assessing sensitivity and heterogeneity across the included studies. *p* < 0.05 was considered to indicate a statistically significant difference. Publication bias was assessed using funnel plots and Egger's test, and *p* < 0.1 was considered to indicate significant publication bias. Statistical heterogeneity was assessed using the inconsistency index (*I*^2^) statistic. Heterogeneity was classified as low, moderate, or substantial if the score was 25, 50, or 75%, respectively. Values >50% were considered to indicate moderate-to-high heterogeneity, and meta-regression and subgroup analysis were performed based on the study design, country, and sample size. In addition, sensitivity analysis was used to identify the study or studies explaining most of the heterogeneity; then, these studies were removed, and the remaining studies were subjected to meta-analysis using a random-effects model. We used Stata (version 15.0) for all statistical analyses.

## 3. Results

### 3.1. Search results and study characteristics

**Figure 1:**
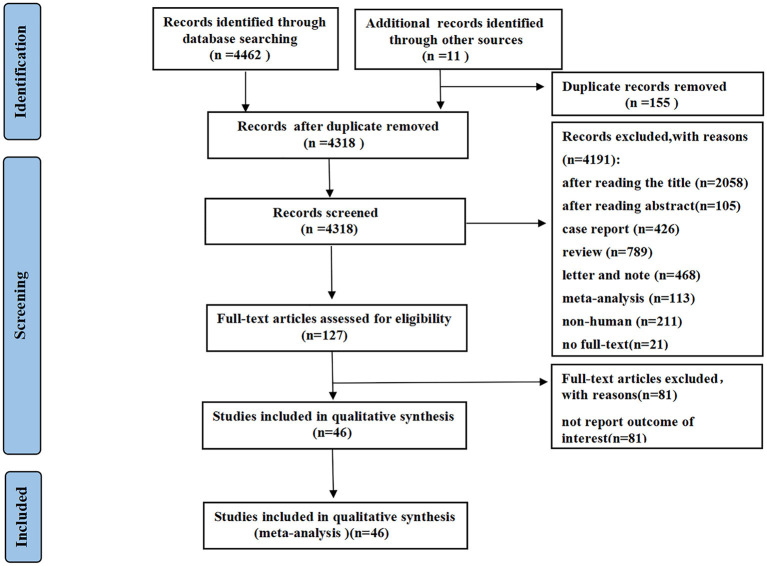
Preferred reporting items for meta-analyses (PRISMA) flow diagram.

A total of 4,473 records were identified using the described search strategy (3,891 from PubMed, 122 from Cochrane Library, 449 from EMBASE, and 11 from other sources). After a detailed assessment based on the inclusion criteria, a total of 46 studies (1–3, 5, 6, 16–56) with 28,663 patients (6,310 in the severe group and 22,353 in the non-severe group) diagnosed with COVID-19 were included in the meta-analysis (Figure 1). The median age of the subjects ranged from 43 to 69 years old. The proportion of males ranged from 42.6 to 81%.

The characteristics of all included studies are presented in Supplementary Table 2. Most studies were retrospective in nature (11 case–control studies and 25 cohort studies), except for 10 prospective studies (2 cross-sectional studies and 8 cohort studies). Six studies reported multicentre data, while the remaining reported single-center data. Thirty-three studies were from China, and the remaining studies were from Japan, Austria, America, France, Spain, Iran, Ethiopia, and Brazil. All studies were published as a full manuscript. All studies received quality scores varying from 6 to 8 points, indicating high quality.

### 3.2. Study outcome

#### 3.2.1. The prevalence of liver injury and baseline chronic liver diseases

**Figure 2:**
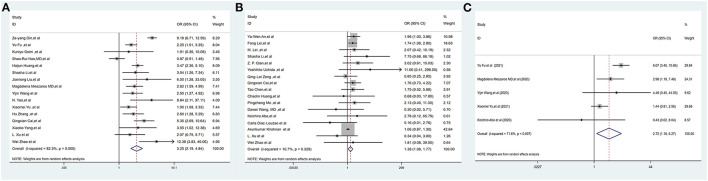
Forest plots of the included studies evaluating abnormal liver chemistries between severe and non-severe patients with COVID-19. **(A)** Prevalence of liver injury. **(B)** The proportion of patients with a history of CLD. **(C)** The mortality association of COVID-19 infection between liver injury and non-liver injury cases. CLD, chronic liver disease.

Liver injury was defined as an elevated transaminase level >3 times the upper limit of normal. Sixteen studies (2, 17, 20, 22–25, 30, 32, 39, 42–44, 46, 55, 56), including 6,041 COVID-19-positive patients, reported the incidence of liver injury. The pooled OR of liver injury was higher in severe COVID-19 patients, with high heterogeneity (OR = 3.25, 95% CI: 2.19–4.84, *I*^2^ = 82.3%, *P*_Heterogenity_ = 0.000) (Figure 2A, Supplementary Table 3).

Seventeen studies (1, 3, 5, 16, 17, 19, 28–30, 33, 35, 36, 45, 50, 52, 55, 56), including 12,138 COVID-19-positive patients, reported the proportion of patients with CLD. The pooled proportion of patients with CLD at baseline was higher in severe COVID-19 cases, with low heterogeneity (OR = 1.38, 95% CI: 1.08–1.77, *I*^2^ = 10.7%, *P*_Heterogenity_ = 0.329) (Figure 2B, Supplementary Table 3).

#### 3.2.2. Serum alanine aminotransferase (ALT)

**Figure 3:**
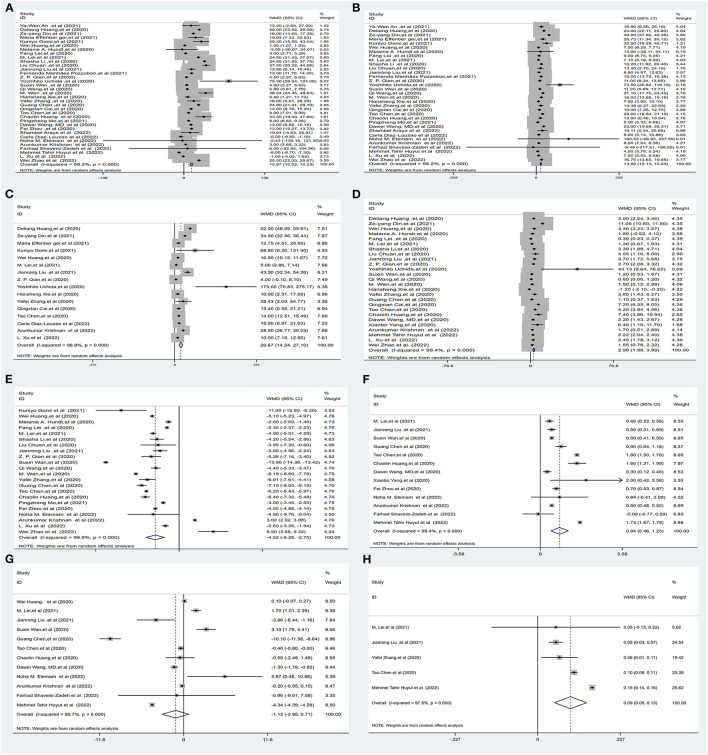
Forest plot showing the mean serum weight levels of liver chemistries between severe and non-severe patients with COVID-19. **(A)** Serum ALT levels. **(B)** Serum AST levels. **(C)** Serum GGT levels. **(D)** Serum TBIL levels. **(E)** Serum ALB levels. **(F)** Serum PT levels. **(G)** Serum APTT levels. **(H)** INR. ALB, albumin; PT, prothrombin time; APTT, activated partial thromboplastin time; INR, international normalized ratio.

Thirty-five studies (1, 5, 6, 16–21, 23, 26–38, 40, 41, 47–56) (25,596 subjects) reported outcome data on ALT. More COVID-19 patients had severe disease than non-severe disease, and the pooled WMD was higher than the mean levels of ALT [(WMD: 12.87U/L; 95% CI: 10.52–15.23); *I*^2^ = 99.2%, *P*_Heterogenity_ = 0.000] (Figure 3A, Supplementary Table 3).

#### 3.2.3. Serum aspartate aminotransferase (AST)

Thirty-three studies (1, 5, 6, 16, 17, 19–21, 23, 26–38, 40, 41, 47, 49–56) (25,384 subjects) reported outcome data on AST. The pooled WMD of AST levels was higher in severe cases, with high heterogeneity (WMD = 13.98 U/L, 95% CI: 12.13–15.83, *I*^2^ = 98.2%, *P*_Heterogenity_ = 0.000) (Figure 3B, Supplementary Table 3).

#### 3.2.4. Serum aspartate gamma-glutamyl transferase (GGT)

Sixteen studies (6, 17, 19–21, 23, 26, 29, 32, 35, 36, 41, 47, 50, 52, 55) (10,944 subjects) reported outcome data on GGT. The mean level of GGT was significantly higher in severe groups, with high heterogeneity (WMD = 20.67 U/L, 95% CI: 14.24–27.10, *I*^2^ = 98.8%, *P*_Heterogenity_ = 0.000) (Figure 3C, Supplementary Table 3).

#### 3.2.5. Serum total bilirubin (TBIL)

Twenty-six studies (1, 5, 6, 17–20, 26–32, 35–38, 40–42, 47, 52, 54–56) (23,571 subjects) reported outcome data on serum bilirubin TBIL. The mean level of bilirubin was higher in the severe COVID-19 group than in the non-severe group (WMD = 2.98 μmol/L, 95% CI: 1.98–3.99), with high heterogeneity (*I*^2^ = 99.4%, *P*_Heterogenity_ = 0.000) (Figure 3D, Supplementary Table 3).

#### 3.2.6. Serum albumin (ALB)

Twenty-two studies (5, 18, 19, 23, 26–33, 35, 37, 38, 40, 47, 48, 51, 52, 55, 56) (16,574 subjects) reported the mean level of serum albumin ALB. The mean level of albumin was lower in the severe COVID-19 group than in the non-severe group (WMD = −4.52 g/L, 95% CI: −6.28 to −2.75), with high heterogeneity (*I*^2^ = 99.9%, *P*_Heterogenity_ = 0.000) (Figure 3E, Supplementary Table 3).

#### 3.2.7. Serum prothrombin time (PT), activated partial thromboplastin time (APTT), and international normalized ratio (INR)

Thirteen studies (1, 5, 18, 19, 26, 29, 32, 37, 42, 48, 51–54) (9,739 subjects) reported the mean level of PT. The mean level of PT was significantly higher in severe cases (WMD = 0.84 s, 95% CI: 0.46–1.23), with high heterogeneity (*I*^2^ = 99.4%, *P*_Heterogenity_ = 0.000) (Figure 3F, Supplementary Table 3).

Twelve studies (1, 5, 18, 19, 26, 29, 32, 37, 51–54) (12,119 subjects) reported the mean APTT. The mean level of APTT had no statistical significance between the severe COVID-19 and non-severe groups (WMD = −1.12, 95% CI: −2.95 to 0.71), with high heterogeneity (*I*^2^ = 99.7%, *P*_Heterogenity_ = 0.000) (Figure 3G, Supplementary Table 3).

Five studies (19, 29, 32, 47, 54) (5,332 subjects) reported the international normalized ratio. The INR was significantly higher in severe cases (WMD = 0.09, 95% CI: 0.05–0.13), with high heterogeneity (*I*^2^ = 97.6%, *P*_Heterogenity_ = 0.000) (Figure 3H, Supplementary Table 3).

#### 3.2.8. Mortality between liver injury and non-liver injury

Five studies (2, 3, 22, 39, 44) (1,224 subjects) reported outcome data on the mortality between liver injury and non-liver injury. The pooled odds ratio of mortality remained higher among subjects in the liver injury group than in the non-liver injury group, with moderate heterogeneity (OR = 2.72, 95% CI: 1.18–6.27, *I*^2^ = 71.6%, *P*_Heterogenity_ = 0.007) (Figure 2C, Supplementary Table 3).

#### 3.2.9. The relationship between drugs and liver injury

Seven studies (16, 22–24, 39, 44) reported liver injury in patients with COVID-19 received antiviral drugs, such as lopinavir/ritonavir. The percentage of patients receiving lopinavir or ritonavir treatment was markedly higher in patients with liver injury with high heterogeneity (OR = 2.09, 95% CI: 1.07–4.08, *I*^2^ = 78.5%, *P*_Heterogenity_ = 0.000) (Supplementary Figure 1A, Supplementary Table 3). Five studies (16, 22, 23, 39, 44) reported liver injury in patients with COVID-19 received antibiotics drugs, such as cephalosporins. The percentage of patients receiving antibiotics treatment was markedly higher in patients with liver injury with high heterogeneity (OR = 2.46, 95% CI: 0.98–6.18, *I*^2^ = 89.1%, *P*_Heterogenity_ = 0.000) (Supplementary Figure 1B, Supplementary Table 3). In addition, six studies (3, 16, 22–24, 44) reported liver injury in patients with COVID-19 received corticosteroid drugs. The percentage of patients receiving corticosteroid treatment was markedly higher in patients with liver injury with moderate, heterogeneity (OR = 1.64, 95% CI: 1.07–2.53, *I*^2^ = 65%, *P*_Heterogenity_ = 0.014) (Supplementary Figure 1C, Supplementary Table 3).

### 3.3. Heterogeneity

There was significant heterogeneity in our results. However, a meta-regression analysis and subgroup analysis based on study type, country, and sample size could not identify the source of heterogeneity (Supplementary Table 4).

### 3.4. Sensitivity analysis

**Figure 4:**
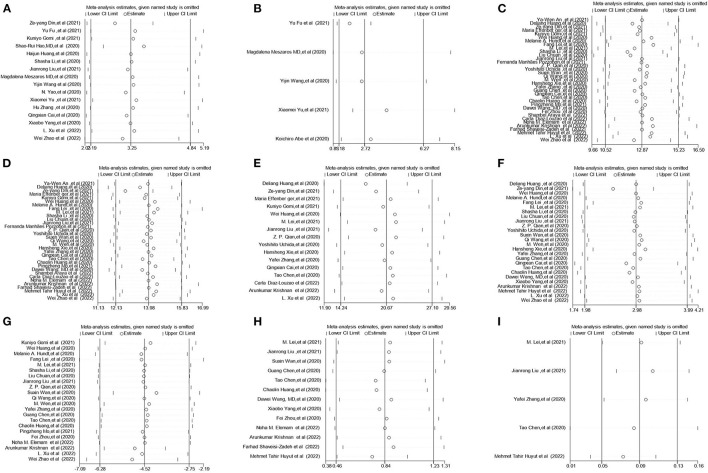
Sensitivity analysis of the serum liver chemistries between severe and non-severe patients with COVID-19. **(A)** Prevalence of liver injury. **(B)** The mortality association with COVID-19 infection between liver injury and non-liver injury cases. **(C)** Serum ALT levels. **(D)** Serum AST levels. **(E)** Serum GGT levels. **(F)** Serum TBIL levels. **(G)** Serum ALB levels. **(H)** Serum PT levels. **(I)** INR levels. ALT, alanine aminotransferase; AST, aspartate aminotransferase; GGT, gamma-glutamyl transferase; TBIL, total bilirubin; ALB, albumin; PT, prothrombin time; INR, international normalized ratio.

In the sensitivity analysis, one individual study was removed at a time and the remaining studies were analyzed to assess whether the excluded study had a dominant effect on the overall pooled results. We found that the results did not change substantially (Figure 4, Supplementary Figure 1), which demonstrated the reliability of our original meta-analysis.

### 3.5. Publication bias

**Figure 5:**
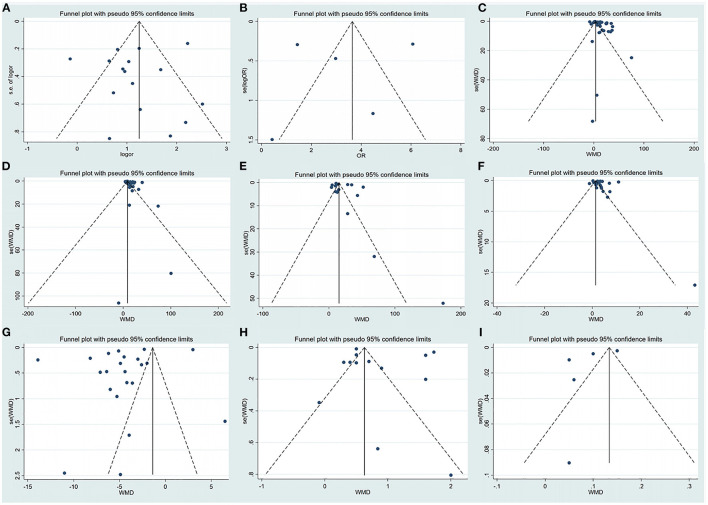
Funnel plot of the included studies evaluating abnormal liver chemistries between severe and non-severe patients with COVID-19. **(A)** Prevalence of liver injury. **(B)** The mortality association with COVID-19 infection between liver injury and non-liver injury cases. **(C)** Serum ALT levels. **(D)** Serum AST levels. **(E)** Serum GGT levels. **(F)** Serum TBIL levels. **(G)** Serum ALB levels. **(H)** Serum PT levels. **(I)** INR levels. ALT, alanine aminotransferase; AST, aspartate aminotransferase; GGT, gamma-glutamyl transferase; TBIL, total bilirubin; ALB, albumin; PT, prothrombin time; INR, international normalized ratio.

In this analysis, there was no evidence of publication bias based on visual inspection of the funnel plot and quantification of publication bias by Egger's test (Table 1, Figure 5; Supplementary Figure 1).

**Table 1:** Egger's test of the studies included in the meta-analysis.

**Outcome**	**Std. eff (bias)**	**Coef**.	**Std. err**.	***t***	***P* > |*t*|**	**[95% conf. interval]**
Liver injury	Bias	−0.73	−1.35	−0.55	0.594	(−3.62, 2.15)
ALT level	Bias	6.03	2.09	2.89	0.007	(1.78, 10.28)
AST level	Bias	3.52	1.47	2.39	0.023	(12.13, 15.83)
GGT level	Bias	3.47	3.16	1.10	0.290	(−3.30, 10.24)
TBIL level	Bias	6.05	4.34	2.09	0.047	(0.084, 12.02)
ALB level	Bias	−13.15	8.02	−1.64	0.318	(−5.01, 14.10)
PT level	Bias	4.54	4.34	1.05	0.318	(−5.01, 14.10)
INR	Bias	−5.72	3.72	−1.54	0.222	(−17.56, 6.13)
Mortality	Bias	−1.57	6.00	−0.26	0.810	(−20.64, 17.50)
Antiviral	Bias	2.55	1.56	1.63	0.177	(−1.78, 6.89)
Antibiotics	Bias	8.38	3.09	2.71	0.073	(−1.45, 18.22)
Corticosteroid	Bias	1.73	1.78	0.97	0.388	(−3.23, 6.69)

CLD, chronic liver disease; ALT, alanine aminotransferase; AST, aspartate aminotransferase; GGT, gamma-glutamyl transferase; TBIL, total bilirubin; ALB, albumin; PT, prothrombin time; APTT, activated partial thromboplastin time; INR, international normalized ratio.

## 4. Discussion

The overall result of this meta-analysis is that the extent of liver injury may depend largely on the severity of COVID-19. In this study, we found that the severity and mortality risk of COVID-19 are related to liver injury, and this study confirms that liver injury exists in COVID-19 patients. Liver injury not only damages the liver but also affects the synthesis and metabolism of factors related to liver function (7). COVID-19-associated liver injury is defined as any liver damage or synthetic or metabolic function impairment in COVID-19 patients with or without a history of liver disease (7).

To our knowledge, our meta-analysis is the first to summarize liver enzymology, ALB synthesis, coagulation function, and TBIL excretion simultaneously based on three major functions of the liver. Previously, liver injury was reported to be an important risk factor for the severity of disease and mortality in SARS and MERS. Our results were consistent with previous reviews (8, 57, 58).

### 4.1. Hepatocellular injury and COVID-19

Liver damage or hepatocellular injury, according to a recent report, was mainly characterized by liver dysfunction, including abnormal levels of ALT, AST, and GGT. Furthermore, it is positively correlated with the severity of COVID-19. These research results were consistent with those of Ding et al. (20) and Lei et al. (29).

The present study shows that the serum levels of ALT, AST, and GGT were significantly increased in severe cases, supporting previous studies reporting damage to the liver parenchyma leading to lobular inflammation and hepatocyte apoptosis (59). Based on our analysis, the pooled WMDs of ALT, AST, and GGT were 12.87, 13.98, and 20.67 U/L, respectively, among severe cases. The serum level of AST and GGT significantly elevated than ALT. AST reflects the destruction and injury of hepatocytes, and GGT, as a “Cholangiocyte-associated enzymes,” reflects the damage of the bile duct. At the same time, it is a great significance for the evaluation of drug-induced liver damage. Focusing on the monitoring of AST and GGT are important for the screening of severely affected patients at early disease stages. This is consistent with a study that GGT was identified as the most sensitive early parameter in severe COVID-19 patients (60). In this study, we found that the proportion of patients with a history of CLD was significantly larger in severe cases, and patients with CLD may have elevated liver indicators at baseline. This virus and the current pandemic limit the number of comparative studies that can be performed at this time.

### 4.2. Liver metabolism injury and COVID-19

Patients with COVID-19 show abnormal levels of indicators of liver metabolism (TBIL). Our meta-analysis showed that the serum levels of TBIL concentrations were significantly increased in severe cases. In a recent study by Xu et al. (59), liver biopsy was performed and showed mild lobular and portal vein activity with moderate microvascular steatosis, possibly due to elevated TBIL, in patients with COVID-19.

### 4.3. Liver synthetic function damage and COVID-19

Moreover, SARS-CoV-2 virus infection affects the synthesis of factors related to liver function (serum albumin and PT). Our meta-analysis showed that elevated PT levels were accompanied by slightly decreased ALB levels in severe cases. However, there was no significant difference in the APTT between severe and non-severe cases. As previously demonstrated, disease severity and death strongly correlate with low serum ALB at admission. Our results are consistent with the findings of these previous studies (5, 23, 42).

Several pathogenic mechanisms could explain the decrease in albumin in patients with COVID-19: (i) gastrointestinal absorption decreased; (ii) disease depletion; (iii) transcapillary leakage: under inflammatory conditions, albumin meridians leak from the microvessel due to the capillary osmotic pressure increases. This disruption of endothelial integrity leads to albumin leakage into the pulmonary interstitium (61); and (iv) COVID-19-related renal injury with disruption of the glomerular filtration barrier, which leads to increased permeability to albumin and its leakage into urine. Decreased albumin may lead to immune function impairment. Consequently, the serum albumin concentration decreases in many critically ill patients.

Accumulated evidence has revealed that coagulation disorder often occurs in COVID-19, with a higher incidence in severe cases. Prolongation of the PT is relatively uncommon in COVID-19, and the mechanisms of coagulopathy are uncertain. Inflammatory cytokines, lymphocyte death, hypoxia, and endothelial cell injury may be implicated in the coagulopathy of patients with COVID-19 (9).

Currently, the mechanisms of COVID-19 associated with liver injury are unclear. Several potential mechanisms have been hypothesized (48, 59). First, previous studies (59) have shown increased serum concentrations of proinflammatory cytokines, including IL-6, IL-1β, and TNF-α, in the majority of severe cases, suggesting that cytokine storm syndrome might be associated with disease severity. This may result in liver injury in patients with COVID-19.

Second, this liver injury may be related to the direct infection of liver cells by viruses (59). COVID-19 enters host cells in the lungs, kidneys and heart *via* angiotensin converting enzyme 2 (ACE2). However, the incidence of liver damage in COVID-19 patients is higher than that of myocardial damage and renal damage. Pathological examination revealed significant damage in the liver but not in the heart tissue. ACE2, as the binding site, is expressed at extremely low levels in hepatocytes and mainly in bile duct epithelial cells (62), which play important roles in the initiation and regulation of immune responses and liver regeneration. Moreover, liver biopsy has revealed mild lobular and portal vein activity with moderate duct involvement or multiorgan failure in patients with SARS-CoV-2 virus infection, as well as microvascular steatosis, possibly due to SARS-CoV-2 virus infection or drug-induced liver injury (59).

Steroids, antibiotics and antivirals are widely used for the treatment of COVID-19. However, there is not enough evidence to demonstrate that currently available drug combinations impair liver function in COVID-19 patients (42). Hao et al. (24) showed that liver injury might be caused by lopinavir/ritonavir, which is used as an antiviral for the treatment of COVID-19 patients. In other studies, An et al. (16) and Fu et al. (22) observed significant liver injury in severe cases with COVID-19 treated with antivirals and antibiotics. This was consistent with our findings.

Collectively, despite all these explanations, the mechanism of COVID-19-associated liver injury remains unclear and needs further research. Nevertheless, our meta-analysis has several strengths. To the best of our knowledge, this is the first comprehensive description of the association of liver injury with COVID-19 in terms of hepatocellular injury and liver metabolic and synthetic function impairment. Additionally, coagulopathy due to COVID-19 and the relationship between COVID-19-related mortality and liver injury were also investigated. In contrast, earlier meta-analyses (8, 57, 58) have mostly focused on the proportion of COVID-19 patients with abnormal liver enzyme levels.

This meta-analysis also has some limitations. First, there was moderate to high heterogeneity in this study. Meta-regression and subgroup analyses were conducted but did not reveal the cause of heterogeneity. The current considerations are that most of the included studies were cohort studies, the sample sizes of some subgroups were small, and incomplete information was available. Second, there is a lack of reports on liver failure occurring in COVID-19 patients and reporting including baseline liver enzyme data in patients with CLD before admission. Third, we did not perform a subgroup analysis for sex, for age, or drug treatments.

## 5. Conclusion

In summary, according to this analysis of existing evidence, while COVID-19-associated liver injury is generally mild, it is more obvious in severe cases, as assessed by serum markers (ALT, AST, GGT, TBIL, ALB, PT, and INR levels), especially AST, GGT, TBIL, ALB, and PT. Hepatocellular injury, liver metabolic, and synthetic function abnormality were observed in severe COVID-19. Liver injury can affect the severity and mortality risk of COVID-19. From a clinical perspective, liver biochemical parameters could be biomarkers for the screening of severely affected patients at early disease stages, and COVID-19 patients should be more closely monitored for the occurrence of liver injury.

## Data availability statement

The original contributions presented in the study are included in the article/Supplementary material, further inquiries can be directed to the corresponding author.

## Author contributions

HW, XZ, and PJ collected and analyzed the data. JF resolved disagreements in case of any conflict. RY designed the study and wrote the first draft of the manuscript. JZ designed and supervised the study and finalized the manuscript. All authors have read and agreed to the published version of the manuscript.
