# Supplementary material for: Liver injury associated with the severity of COVID-19: A meta-analysis
Source: Front Public Health. 2023 Feb 2;11:1003352. doi: 10.3389/fpubh.2023.1003352 (PMC9932800; doi:10.3389/fpubh.2023.1003352)
Supplement: Supplementary file 1 [file Data_Sheet_1.pdf]

Supplementary Table 1 Search strategy detailed in the meta-analysis.

Electronic database	Search strategy	Results(n)
PubMed	(((((hepatitis)[Title/Abstract] OR (liver disease))[Title/Abstract] OR (acute Liver Injury))[Title/Abstract] OR ((liver Injury) [Title/Abstract] AND (2019:2022[pdat])) AND (((covid-19) [Title/Abstract] OR (SARS-CoV-2))[Title/Abstract] OR (coronavirus) [Title/Abstract] AND (2019:2022[pdat]))) AND (((Pneumonia)[Title/Abstract] OR (Pulmonary Inflammation))[Title/Abstract] OR (Pneumonitis) [Title/Abstract] AND (2019:2022[pdat]))	3891
Embase	[('coronavirus disease 2019'/exp) OR ('sars-cov-2':ab,ti) OR ('coronavirus':ab,ti)]AND [（'pneumonia'/exp）OR（Pneumonitis）OR（Pulmonary Inflammation）]AND[（hepatitis）OR(liver disease) OR (liver Injury)]	449
Cochrane Library	(((((liver Injury) OR (hepatitis)) OR (liver disease)) OR (acute Liver Injury)) AND (((covid-19) OR (SARS-CoV-2)) OR (coronavirus))) AND (((Pneumonia) OR (Pulmonary Inflammation)) OR (Pneumonitis))	122

Supplementary Table 2 Characteristics of the included studies in the meta-analysis.

First author (year)	Coun-try	Study design	Samples (N)	Sex N (%)	Mean（SD） Age (year)	Liver Function Test Mean (SD) or N %				Death,N(%)			Study Quality
							Total	Severe Group	Nonsevere Group	Total	Liver injury	Non -injury	
Koichiro Abel （2020）	Japan	RS	Total: 22	M: 13 (59)	47.8（5.3）	NA	NA	NA	NA	2 (9)	1 (7)	1 (14)	6
			Severe:2	F:9 (41)		NA	NA	NA	NA				
			Nonsevere: 20			NA	NA	NA	NA				
Ya-Wen An （2021）	China	PS	Total: 253	M: 113(44.7)	45.3(17.8)	ALT:	34.6(49.4)	44.2(51.0)	32.0(48.8)	NA	NA	NA	7
			Severe:58	F:140 (55.3)		AST:	31.1(25.0)	43.6(34.3)	27.8(20.9)				
			Nonsevere: 195										
Deliang Huang （2020）	China	PS	Total: 318	M: 198 (62.3)	NA	ALT:	46(8.2)	67(8.8)	41(7)	NA	NA	NA	8
			Severe:85	F: 130（37.7）		AST:	38（4)	58(8.7)	34(3)				
			Nonsevere: 233			GGT:	47.5(9.2)	92(18)	40(6.2）				
						TBIL:	20(2.2)	22(1.7)	19(2.2)				
Ze-yang Din （2021）	China	RS	Total: 2073	M:1024 (49.4)	62(3.3)	Live injury:	297 (14.3%)	103 (51.5%)	94 (10.4%)	200(9.6)	185 (92.5)	15(7.5)	8
			Severe:200	F: 1049(50.6)		ALT:	33(6.3)	48(9.5)	48(9.5)				
			Nonsevere: 1873			AST:	30(4.5)	68(16.9)	68(16.9)				
						TBIL:	11.4(1.3)	22.05(3.2)	11(1.1)				
						GGT:	37（7.8）	69.5（13.8）	35（7.2）				
Yu Fu (2020)	China	RS	Total: 482	M:243 (50.4)	56.5(4.7)	Live injury:	142(29.5%)	75(39.9%)	67 (22.8%)	64 (13.3)	42 (29.6)	22 (6.5)	6
			Severe:188	F: 239（49.6）									
			Nonsevere: 294										

Supplementary Table 2 (continued)

Kuniyo Gomi (2021)	Japan	RS	Total: 210	M:119(56.7)	49(21)	Live injury:	124(59%)	5（71.4%）	119(58.6%)	NA	NA	NA	7
			Severe:7	F: 91(43.3)		ALT:	27(26.8)	55.3(17.9)	26(26.5)				
			Nonsevere:203			AST:	30.1(28)	61.5(18.5)	29(27.8)				
						GGT:	33.3(53.3)	99.8(84)	31(50.7)				
						ALB:	38.6(6)	28(6.4)	39(5.7)				
Shao-Rui Hao,MD (2021)	China	RS	Total:788	M:407(51.6)	NA	Live injury：	222(28.2%)	20（25.6%）	202(28.5)	NA	NA	NA	6
			Severe:78	F: 381 (48.4)									
			Nonsevere：710										
Haijun Huang (2021)	China	RS	Total:675	M: 313(46.3)	55(4.8)	Live injury：	305(45.2%)	104（68.4%）	201(38.4)	NA	NA	NA	6
			Severe:152	F: 362(53.9)									
			Nonsevere:523										
Wei Huang (2020)	China	RS	Total:2623	M:1323（50.4）	62.3(4.2)	ALT:	24.4(4.5)	25.5(4.9)	24(4.3)	NA	NA	NA	7
			Severe:615	F:1300（49.6）		AST:	25.6(5.7)	31(8.8)	24(2.7)				
			Nonsevere:2008			TBIL:	9(1.9)	11.7(2.1)	8.3(0.9)				
						GGT:	31.6(7.8)	39.9(9.3)	29(5)				
						ALB:	34.3(2.5)	30.4(1.6)	35.5(1.01)				
						PT:	13.8(0.8)	14.9(1.1)	13.5(0.15)				
						APTT:	38.6(1.4)	38.7(2.1)	38.6(1.1)				
Melanie A. Hundt (2020)	Network	RS	Total:1827	M: 969 (53.0)	64.6(18.2)	ALT:	29(304.3)	28(304.3)	31(239.2)	NA	NA	NA	7
			Severe:652	F: 858(47)		AST:	42(507.7)	51(507)	38(231.8)				
			Nonsevere:1175			TBIL:	8.6(25.1)	8.6(25.1）	6.8(22.5)				
						ALB:	35(7)	33(6)	35(6.8)				

Supplementary Table 2 (continued)

Fang Lei	China	RS	Total:5771	M:2724 (47.2)	56.5(3.7)	CLD%:	81 (1.4%)	25 (2.1%)	56 (1.2%)	NA	NA	NA	7
(2020)			Severe:1186	F: 3047（52.8）		ALT:	24(4.0)	26(4.7)	23(3.8)				
			Nonsevere:4585			AST:	24(3)	31(4.2)	22(2.3)				
						TBIL:	10.4(1)	10.6(1.2)	10.3(1)				
						ALB:	37.5(1.1)	35.7(1.1)	38(1.03)				
M. Lei	China	RS	Total:288	M: 131 (45.5)	43.9（8.3）	ALT:	22.5（3.4）	45.8（8.8）	21.3（3）	NA	NA	NA	8
N.			Severe:32	F: 157（54.5）		AST:	18.4(1.8)	24.8(5.5)	17.7(1.7)				
(2021)			Severe:32			TBIL:	9.8(1.7)	10.7(1.8)	9.4(0.9)				
						GGT:	17(5.8)	21(5.7)	16(6.8)				
						ALB:	37.1(2.7)	34.7(1.5)	39.6(2.6)				
						PT:	13.5(0.45)	13.6(0.5)	13.2(0.5)				
						APTT:	35.1(1.6)	36.6(1.9)	34.9(1.7)				
						INR:	1.01(0.02)	1.03(0.5)	0.98(0.3)				
						CLD%:	10 (3.5%)	2 (6.3%)	8 (3.1%)				
Shasha Li(2020)	China	PS	Total: 159	M:90（56.6）	38.5(4.9)	Live injury：	28(17.6%)	11 (32.4%)	17 (13.6%)	NA	NA	NA	7
			Severe:34	F:69（43.4）		ALT:	27 (7.3)	57.6(8.2)	22.75(5.2)				
			Nonsevere:125			AST:	24.8(3.3)	39.8(12.7)	23.6(1.9)				
						TBIL:	16.7(3.1)	19.3(4)	16(2.4)				
						ALB:	39(2)	35.5(3.8)	39.7(2.4)				
						CLD%:	3(1.9%)	2(5.9%)	1(0.8%)				
Liu	China	RS	Total: 32	M:20（62.5）	38.6(5)	ALT:	27 (7.3)	60.25(7)	22.8(5.2)	NA	NA	NA	8
Chuan			Severe:4	F:12（37.5）		AST:	24.75(3.3)	37(10.9)	23.6(1.9)				
(2020)			Nonsevere:28			TBIL:	16.5(2.9)	20.5(3.4)	15.95(2.4)				
						ALB:	39.5（2.5）	35.75（3.3）	39.7(2.4)				

Supplementary Table 2 (continued)

Jianrong Liu (2021)	China	RS	Total:58	M:30 (51.7)	65.1（14.2）	Live injury：	30（51.7%）	10 (33.3%)	20（43.6%）	NA	NA	NA	8
			Severe:12	F:28（48.3）		ALT：	21.6（8.9）	31.8（11.4）	19（5.8）				
			Nonsevere:46			AST：	22.8（5）	29.8（6.7）	21（2）				
						TBIL：	9.4（2.6）	12.3（3.4）	8.6（1.6 ）				
						GGT:	32(20.6)	66.3(19)	23(7.3)				
						ALB:	37.1(2.2)	34.2(2.3)	37.8(1.4)				
						PT:	13.4(0.4)	13.8(0.3)	13.3(0.3)				
						APTT：	37.8(2.9)	34.8(4.6)	38.6(1.5)				
						INR:	1.03(0.04)	1.07(0.03)	1.02(0.03)				
Magdalena Meszaros (2020)	France	RS	Total:234	M:149 (63.7)	67(14)	Live injury	156(66.7%)	89（78.1%）	67(55.8)	37 (15.8)	31(19.9)	6 (7.7)	6
			Severe:114	F:85（36.3）									
			Nonsevere:120										
Fernanda Manhães Pozzobon(2021)	Brazil	PS	Total:406	M:231 (57)	56(3.3)	ALT：	38(6.2)	48(7)	35(5.3)	NA	NA	NA	
			Severe:149	F:175（43）		AST:	37(5)	49(7.5)	34(3.5)				
			Nonsevere:257										
Z.P. Qian (2020)	China	RS	Total:324	M:167(51.5)	51(12.2)	ALT:	21.86(3.14)	26(3.9)	21.5(2.8)	NA	NA	NA	6
			Severe:26	F:157(48.5)		AST:	23.9(4.13)	34（6.9）	23(2.2)				
			Nonsevere:298			TBIL:	8.3(1.1)	10.75(1.6)	8.05(0.7)				
						GGT:	24.3(5.18)	28(10.6)	24(4.3)				
						ALB:	40.6(4.13)	35.79(4.75)	41.07(3.8)				
						CLD%:	20(6.2%)	2(7.7%)	18(6%)				

Supplementary Table 2 (continued)

Fei Zhou	China	RS	Total:191	M:119 (62)	56(3.5)	ALT:	30(4.8)	40(0.8)	27(4.2)	NA	NA	NA	6
(2020)			Severe:54	F:72 (38)		ALB:	32.3(1)	29.1(1.2)	33.6(1)				
			Nonsevere:137			PT:	11.6(0.4)	12.1(0.6)	11.4(0.4)				
Yoshihit	China	RS	Total:35	M:22 (62.9)	52(15.75)	ALT:	38(57.25)	104.3(64.3)	29(52.5)	NA	NA	NA	6
o Uchida			Severe:8	F:13 (37.1))		AST:	34(51.5)	104.5(56.4)	31(45.5)				
(2020)			Nonsevere:27			TBIL:	12(39.8)	53.43(48.2)	10.3(6.4)				
						GGT:	37(121)	203(140)	30(85)				
Suxin	China	PS	Total:135	M:72 (53.3)	47(3.2)	ALT:	26(3.38)	26.6(4.7)	21.7(3.68)	NA	NA	NA	7
Wan			Severe:40	F: 63 (46.7)		AST:	33.4(2.65)	33.6(4.63)	22.4(2.3)				
(2020)			Nonsevere:95			TBIL:	8.6(1.3)	9.8(1.95)	8.6(1.4)				
						ALB:	40.5(1.07)	36(1.38)	49.9(1.03)				
						PT:	10.9(0.15)	11.3(0.28)	10.8(0.15)				
						APTT:	26.9(0.72)	29.7(4.2)	26.6(0.72)				
						CLD%:	2 (1.5%)	1 (2.5%)	1 (1%)				
Yijin	China	PS	Total:156	M:82(52.6)	51.2(16)	Live	64(42%)	30（55.6%）	34（33.3%)	4(2.6)	3 (4.7)	1 (1.1)	6
Wang						injury							
(2020)			Severe:54	F:74(47.4)									
			Nonsevere:102	M:56（53.3)									
Qi Wang	China	RS	Total:105	F:49（46.7）	45(4.3)	ALT:	23.5(3.7)	27.8(4.8)	22(3.4)	NA	NA	NA	8
(2020)			Severe:26			AST:	24.2(3.8)	43.1(8.5)	22(3.3)				
			Nonsevere:79			TBIL:	10.2(1.4)	10.6(1.3)	10(1.5)				
						ALB:	41.6(1.7)	37.6(2.2)	42(1.7)				
M. Wen	China	RS	Total:110	M:59（53.6）	NA	ALT:	34.15(15.3)	66.04(23)	29.5(4.8)	NA	NA	NA	8
(2020)			Severe:14	F:51（46.4）		AST:	31.89(6.3)	45.87(5.9)	29.85(2.7)				
			Nonsevere:96			TBIL	16.7(2.1)	18.05(2.52)	16.55(2)				
						ALB:	35.8(2.8)	28.57(0.7)	36.76(0.94)				

Supplementary Table 2 (continued)

Hanshen g Xie (2020)	China	RS	Total:79<br>Severe:28<br>Nonsevere:51	M:44(55.7)<br>F:35(44.3)	60(3)	ALT:<br>AST:<br>TBIL:<br>GGT:	34(8.2)<br>30(4.5)<br>13.6(1.5)<br>31.5(10.4)	36.5(13.5)<br>35(7.5)<br>12.7(1.83)<br>35.5(14.75)	30(5.63)<br>28(5)<br>13.9(2.45)<br>25.5(19.7)	NA	NA	NA	7
N. Yao (2020)	China	RS	Total:40<br>Severe:22<br>Nonsevere:18	M:24(60)<br>F:16(40)	NA	Live injury:	22(55%)	17(77.3%)	4(22.2%)	NA	NA	NA	6
Xiaomei Yu (2021)	China	RS	Total:330<br>Severe:232<br>Nonsevere:98	M:163(49.4)<br>F:167(50.6	68.3(2)	Live injury:	96(29.1%)	76(32.8%)	20(20.4%)	65（19.7）	23 (24)	42(17.9)	6
Qing-Lei Zeng (2021)	China	RS	Total:70<br>Severe:40<br>Nonsevere:30	M:46 (65.7)<br>F:24（34.3）	56.5(8)	CLD%:	13 (18.6%)	7（17.5%）	6 (20%)	NA	NA	NA	6
Hu Zhang (2020)	China	RS	Total:218<br>Severe:38<br>Nonsevere:180	M:94（43.1）<br>F:124（56.9）	50.1(18.3)	Live injury:	79(36.2%)	21 (55.3%)	58(32.2%)	NA	NA	NA	6
Yafei Zhang (2020)	China	RS	Total:115<br>Severe:31<br>Nonsevere:84	M:49(42.6)<br>F:66(57.4)）	49.5(17.1)	ALT:<br>AST:<br>TBIL:<br>GGT:<br>ALB:<br>INR:	25.7(21.1)<br>28.3(15.7)<br>11.3(5.2)<br>36.1(45）<br>38.8(4.4)<br>1.17(0.1)	37.87(32.17)<br>38.87 (22.55)<br>14.12(6.37)<br>56.90（73.28）<br>34.40(4.11)<br>1.21(0.13)	21.22(12.67)<br>24.39(9.79<br>10.27 (4.26)<br>28.47(24.93）<br>40.41(3.24)<br>1.15(0.09)	NA	NA	NA	7

Supplementary Table 2 (continued)

Guang	China	RS	Total:21	M:17 (81.0)	56.75(3.75)	ALT:	27.5(6.5)	41.6(5.1)	16.8(2.5)	NA	NA	NA	7
Chen			Severe:11	F:4(19)		AST:	30.5(6.5)	49.1(13.5)	24(1.4)				
(2020)			Nonsevere:10			TBIL:	8.7(0.9)	9(0.8)	7.9(0.9)				
						ALB:	33.6(1.95)	30.2(1.3)	30.2(1.3)				
						PT:	13.7(0.4)	14.2(0.3)	13.3(0.3)				
						APTT:	39(2.7)	34.5(1.9)	44.6(1.5)				
Qingxia	China	PS	Total:298	M:145 (48.66)	47.5(4.7)	Live	44 (14.8%)	21 (36.2%)	23 (9.1%)	NA	NA	NA	8
n Ca						injury							
(2020)			Severe：58	F:153(51.34)		CLD%:	28 (9.4%）	8 (13.79%)	20 (8.33%)				
			Nonsevere：240			ALT:	37(3.2)	42(12.5)	30(2.7)				
						AST:	34(3.5)	38(10.6)	28(3)				
						TBIL:	16.2(1.7)	20.7(3.2)	13.5(1.6)				
						GGT:	37.8(3.2)	47(30.3)	33.6(3.5)				
Tao	China	RS	Total:274	M:171 (62.4)	62(4.3)	CLD%:	11 (4%)	6 (5.3%)	5 (3.1%)	NA	NA	NA	8
Chen			Severe113	F:103 (37.6)		ALT:	23(3.8)	28(4.8)	20(2.9)				
(2020)			Nonsevere161			AST:	30(4)	45(6)	25(2.3)				
						TBIL：	9.6(1.13)	12.6(1.2)	8.4(0.9)				
						GGT:	33(5)	42(7.2)	28(4.4)				
						ALB:	33.9(1.2)	30.1(0.9)	36.3(1)				
						PT:	14.3(0.3)	15.5(0.5)	13.9(0.2)				
						APTT:	30.8(1.3)	40.6(1.9)	41(1.2)				
						INR:	1.1(0.03)	1.2(0.05)	1.1(0.02)				
Chaolin	China	PS	Total:41	M:30 (73)	49(4.3)	CLD%:	1 (2%)	0	1 (4%)	6 (15)	5 (38)	1 (4)	8
Huang			Severe:13	F:11 (27)		ALT:	32(7.3)	60.5(25.7)	27(5.1)				
(2020)			Nonsevere:28			AST:	34(5.5)	47(11.7)	34(4.1)				
						TBIL:	11.7(1.1)	18.2(6.5)	10.8(0.7)				
						ALB:	31.4(1.8)	28.3(1.3)	34.7(1.6)				

Supplementary Table 2 (continued)

						PT:	11.1(0.6)	12.3(0.6)	10.7(0.6)				
						APTT:	27(2.5)	27.2(3.3)	27.7(2.3)				
Pingzhe ng Mo (2021)	China	RS	Total:155	M:86 (55.5)	54(4)	CLD%:	7 (4.5%)	5 (5.9%)	2 (2.9%)	NA	NA	NA	7
			Severe:85	F:69(44.5)		ALT:	23(3.7)	28(4.2)	20(4.5)				
			Nonsevere:70			AST:	32(4)	37(6.7)	32(3.8)				
						ALB:	38(1.2)	36(1.3)	39(1.5)				
Dawei Wang (2020)	China	RS	Total:138	M:75 (54.3)	56(4.3)	CLD%:	4 (2.9%)	0	4 (3.9%)	NA	NA	NA	7
			Severe:36	F:63 (45.7)		ALT:	24(4.0)	35(9.5)	23(3.5)				
			Nonsevere:102			AST:	31(4.5)	52(10)	29(2.8)				
						TBIL:	9.8(0.95)	11.5(2.3)	9.3(0.8)				
						PT:	13(0.2)	13.2(0.55)	12.9(0.2)				
						APTT:	31.4(0.7)	30.4(1.4)	31.7(0.7)				
Xiaobo Yang (2020)	China	RS	Total:52	M:35 (67)	59.7(13.3)	Live injury	15 (29%)	9 (28%)	6 (30%)	NA	NA	NA	6
			Severe:20	F:17 (33)		TBIL:	17(10)	12.9(2.9)	13.1(4.3)				
			Nonsevere:32			PT:	12.1(3)		10.9(2.7)				
Maria Effenber ge 2021	Austria	PS	Total: 96	M:60(62.5)	60.7(18.9)	ALT:	28(12.6)	4.93(24.83)	25(4.2)	NA	NA	NA	7
			Severe:15	F:36 (37.5)		AST:	37.2(11.56)	54.7(18.4)	34(5.6)				
			Nonsevere: 81			GGT:	40(10)	50.75(16)	38(7)				
Shambel Araya 2022	Ethiopi a	PS	Total: 440	M:270（61.3）	60.3(1.3)	ALT:	59(63)	59.9(58.7)	49.26(43.9)	NA	NA	NA	7
			Severe:62	F:170（38.7）		AST:	57(53)	66.5(65)	47.39(35.3)				
			Nonsevere: 378										
Carla Diaz-Lo uzao 2022	United States	RS	Total: 440	M:120 (56.60)	68.4(3.43)	CLD%	13(3%)	0	13 (7.4%)	NA	NA	NA	7
			Severe:62	F:97 (43.40）		ALT:	35(6.5)	31(11.5)	36(6.75)				
			Nonsevere: 378			AST:	30(3.8)	37(8.75)	29(3.3)				
						GGT:	45(10)	59(18.25)	44(9)				

Supplementary Table 2 (continued)

Noha M.	United	RS	Total: 440	M:29（78.4）	51.95(14)	ALT:	116.45(200.2)	115.1(226.6)	117.57(180.8)	NA	NA	NA	8
Elemam	States		Severe:62	F:8（21.6）		AST:	133.7(232.3)	188(308.2)	87.47(131.72)				
2022			Nonsevere: 378			ALB:	30.9(7.47)	28.3(9.3)	33.2(4.6)				
						PT:	15.04(1.89)	15.49(2.28)	14.65(1.43)				
						APTT:	42.38(8.19)	45.44(9.27)	39.77(6.25)				
Arunku	United	RS	Total: 3830	M:1959 (51.1)	64.2(4.6)	ALT:	28(4.8)	30(5)	27(4.5)	NA	NA	NA	8
mar	States		Severe:1354	F:1871(48.9)		AST:	36.83(6.3)	42(5.8)	34(4.6)				
Krishnan			Nonsevere: 2476			TBIL:	8.5(16.53)	8.5(18.8)	6.8(16.25)				
2022						GGT:	126.08(25.36)	144.5(31)	116(13.5)				
						ALB:	38.6(1.2)	39(1.2)	36(1.3)				
						PT:	11.1(0.2)	11.4(0.3)	10.9(0.2)				
						APTT:	26.2(5.2)	25.7(5.4)	25.9(5)				
Farhad	Iran	RS	Total: 77	M:35(45.5)	61.6(17.8)	ALT:	77.7(258.8)	82.2(146.98)	76.1(289.1)	NA	NA	NA	7
Shaveisi			Severe:20	F:42(54.5)		AST:	139.5(588.5)	132.5(261.8)	141.98(668.4)				
-Zadeh			Nonsevere: 57			PT:	12.77(1.95)	12.7(0.83)	12.79(2.22)				
2022						APTT:	38(24.7)	37.33(7.2)	38.29(28.52)				
Mehmet	Türkiy	RS	Total: 4597	M:2371(51.6)	57(6.3)	ALT:	31.6(7.6)	24(5.17)	32(7.5)	NA	NA	NA	8
Tahir	e		Severe:233	F:2226(48.4)		AST:	27.7(3.6)	32(5.7)	27.5(3.3)				
Huyut			Nonsevere: 4364			TBIL:	8.5(1)	10.6(1.4)	8.38(0.88)				
2022						PT:	13.2(0.5)	14.83(0.45)	13.1(0.25)				
						APTT:	35.1(1.6)	31(0.3)	35.34(1.31)				
						INR:	1.1(0.04)	1.25(0.04)	1.1(0.02)				
L. Xu	China	RS	Total: 111	M:61（55）	69(3.7)	Live	86 (77.5%)	34 (85%)	52 (73.2%)	NA	NA	NA	8
M. 2022						injury							
			Severe:40	F:50（45）		CLD%:	6 (5.4%）	1 (2.5%)	5 (7%)				
			Nonsevere: 71			ALT:	29.6(4.7)	29(5.9)	30(3.8)				
						AST:	27.7(5.7)	32.5(6.4)	25(2.7)				

Supplementary Table 2 (continued)

						TBIL:	12.2(1.9)	13.75(1.98)	11.3(1.2)				
						GGT:	29.6(7.7)	36(8.75)	26(3.8)				
						ALB:	33.7(1.9)	32(2)	34.6(1)				
Wei	China	RS	Total: 157	M:88（56）	45(18)	Live	20（1.7%）	8 (5.3%)	12 (8.5%)	NA	NA	NA	8
Zhao						injury							
2022			Severe:15	F:69（44）		CLD%:	1.27 (5.4%）	0	1.4 (7%)				
			Nonsevere: 142			ALT:	22.4(8)	45.5(6.23)	20(1.9)				
						AST:	23.6(5.5)	38.75(6.1)	22(1.7)				
						TBIL:	7.8(1.6)	9.25(1.45)	7.7(1.5)				
						ALB:	37(4.8)	43.1(5.4)	36.6(4.3)				

Note: CLD: chronic liver disease; ALT: alanine aminotransferase; AST: aspartate aminotransferase; GGT: gamma-glutamyl transferase; TBIL: total bilirubin; ALB: albumin; PT: prothrombin time; APTT: activated partial thromboplastin time; INR: International Normalized Ratio; RS: retrospective study; PS: prospective study.

Supplementary Table 3 Meta-analysis of liver injury parameters in COVID-19 patients

Parameter	No. studies	No. patients	Heterogeneity P	Heterogeneity I^2	Model	Meta-analysis WMD or OR（95%CI）
Live injury (%) transaminase >3 ULN	16	6041	0.000	82.3%	Random	3.25(2.19,4.84)
CLD%	17	12138	0.329	10.7%	Random	1.38(1.08,1.77)
ALT level（U/L）	35	25596	0.000	99.2%	Random	12.87(10.52,15.23)
AST level（U/L）	33	25384	0.000	98.2%	Random	13.98(12.13,15.83)
GGT level（U/L）	16	10944	0.000	98.8%,	Random	20.67(14.24, 27.10)
TBIL level(μmol/L）	26	23571	0.000	99.4%,	Random	2.98(1.98,3.99)
ALB level（g/L）	22	16574	0.000	99.9%	Random	-4.52(-6.28, -2.75)
PT level（s）	13	9739	0.000	99.4%	Random	0.84(0.46,1.23)
APTT level（s）	12	12119	0.000	99.7%	Random	-1.12(-2.95, 0.71)
INR	5	5332	0.000	97.6%	Random	0.09(0.05, 0.13)
mortality	5	3297	0.007	71.6%	Random	2.718(1.179,6.267)
antiviral	7	1538	0.000	78.5%	Random	2.09(1.07,4.08)
antibiotics	5	790	0.000	89.1%	Random	2.46(0.98,6.18)
corticosteroid	7	564	0.014	65%	Random	1.64(1.07, 2.53)

Note: CLD: chronic liver disease; ALT: alanine aminotransferase; AST: aspartate aminotransferase; GGT: gamma-glutamyl transferase; TBIL: total bilirubin; ALB: albumin; PT: prothrombin time; APTT: activated partial thromboplastin time; INR: International Normalized Ratio.

Supplementary Table 4 Subgroup analyses and meta-analysis regression of included studies in the meta-analysis.

Outcome	Subgroups		No.of studies	Meta-analysis regression (P>|z|)	Effect size (Odds ratio/mean difference)	I^2(%)	P
Liver injury	overall		16	0.548	3.25(2.19,4.84)	82.3%	0.000
	country	China	14	0.819	3.37(2.17, 5.24)	84.5%	0.000
		Abroad	2		2.70(1.57,4.64)	0.0%	0.666
	Study type	cohort study	11	0.938	3.38(1.90,6.00)	87.0%	0.000
		case-control study	5		2.88(2.11,3.94)	20.6%	0.283
	Sample size	≥175	9	0.420	2.83 (1.65,4.84)	89.5%	0.000
		＜175	7		3.88 (2.38,6.32)	31%	0.192
ALT level	overall		35	0.078	12.87(10.52,15.23)	99.2%	0.000
	country	China	24	0.476	14.73(11.92,17.54)	99.1%	0.000
		Abroad	11		7.34(0.88, 13.79)	99.1%	0.000
	Study type	cross-sectional study	2	0.875	21.13(8.20,34.05)	69.5%	0.070
		case-control study	7		17.71(10.48,24.94）	94.7%	0.000
		cohort study	26		11.38(8.83,13.92)	99.3%	0.000
	Sample size	≥175	17	0.015	9.14(6.21,12.06)	99.5%	0.000
		＜175	18		18.41(12.75, 24.07)	97.6%	0.000
AST level	overall		33	0.114	13.98(12.13,15.83)	98.2%	0.000
	country	China	22	0.992	14.40(11.62, 17.18)	98.6%	0.000
		Abroad	11		17.71(8.33,15.09)	95.7%	0.000
	Study type	cross-sectional study	2	0.596	21.22(13.61,28.83)	65.1%	0.090
		case-control study	7		15.26(10.01,20.50)	87.9%	0.000
		cohort study	24		13.11(11.13,15.09)	98.4%	0.000
	Sample size	≥175	16	0.868	14.17(11.65,16.69)	99.0%	0.000
		＜175	17		13.96(10.72,17.20)	91.9%	0.000

Supplementary Table 4 (continued)

GGT level	overall		16	0.025	20.67(14.24, 27.10)	98.8%	0.000
	country	China	11	0.664	19.80(12.47,27.13)	99.0%	0.000
		Abroad	5		23.04(10.62,35.45)	89.8%	0.000
	Study type	cross-sectional study	1	0.022	52.00(48.09,55.91)	.%	-
		cohort study	12		19.32(12.88,25.77)	98.7%	0.000
		case-control study	3		7.96(-1.31,17.24)	65.3%	0.056
	Sample size	≥175	10	0.542	20.46(12.46,28.45)	99.2%	0.000
		＜175	6		21.37(9.57,33.16)	88.8%	0.000
TBIL level	overall		26	0.433	2.98(1.98,3.99)	99.4%	0.000
	country	China	22	0.427	3.12(1.90,4.33)	99.5%	0.000
		Abroad	4		2.03(0.98, 3.08)	54.4%	0.087
	Study type	cross-sectional study	1	0.120	3.00(2.54, 3.46)	.%	-
		cohort study	16		3.40(2.20,4.6)	99.5%	0.000
		case-control study	9		1.31(-0.27, 2.90)	92.6%	0.000
	Sample size	≥175	11	0.199	3.56(2.00,5.12)	99.7%	0.000
		＜175	15		2.05(1.29,2.81)	84.4%	0.000
ALB level	overall		22	0.113	-4.52(-6.28, -2.75)	99.9%	0.000
	country	China	18	0.531	-4.87(-6.16,-3.58)	99.6%	0.000
		Abroad	4		-2.98(-6.95, 1.00)	99.9%	0.000
	Study type	cohort study	17	0.266	-4.00(-5.97,-2.03)	99.9%	0.000
		case-control study	5		-6.28(-8.70,-3.87)	93.9%	0.000
	Sample size	≥175	9	0.864	-4.02(-6.60, -1.45)	100.0%	0.000
		＜175	13		-4.83(-7.34,-2.33)	99.2%	0.000

Supplementary Table 4 (continued)

PT level	overall		13	0.288	0.84(0.46,1.23)	99.4%	0.000
	Study type	retrospective study	11	0.447	0.81(0.35,1.27)	99.5%	0.000
		prospective study	2		1.03(-0.04,2.11)	96.5%	0.000
	Sample size	≥175	5	0.311	0.99(0.30,1.67)	99.8%	0.000
		＜175	8		0.68(0.42,0.94)	85.8%	0.000
	country	China	9	0.861	0.86(0.46,1.25)	97.9%	0.000
		Abroad	4		0.77(-0.14,1.67)	99.8%	0.000
INR	overall		5	0.000	0.09(0.05, 0.13)	97.6%	0.000
	Sample size	≥175	3	0.000	0.12(0.07,0.17)	97.5%	0.000
		＜175	2		0.05(0.03,0.07)	0.0%	0.712
	country	China	4	0.000	0.07(0.03,0.11)	86.7%	0.000
		Abroad	1		0.15(0.14, 0.16)	-	-
Mortality	overall		5	0.989	2.72(1.18,6.27)	71.6%	0.007
	country	China	3	0.727	3.18(0.97,10.44)	83.9%	0.002
		Abroad	2		1.90(0.38, 9.44)	34.6%	0.216
	Study type	cohort study	4	0.322	1.82(1.07, 3.10)	8.4%	0.351
		case-control study	1		6.07(3.46,10.66)	-	-
	Sample size	≥175	3	0.902	2.97(1.14 ,7.70)	83.7%	0.002
		＜175	2		1.67(0.17, 16.21)	34.8%	0.215
antiviral	overall		7	0.048	2.09(1.07,4.08)	78.5%	0.000
	country	China	6	0.671	3.59 (1.64, 7.88)	77.9%	0.001
		Abroad	1		1.85 (0.89, 3.87)	.%	-
	Study type	cohort study	4	0.000	1.14 (0.78, 1.67)	18.0%	0.301
		case-control study	3		4.30 (2.50, 7.38)	0%	0.527
	Sample size	≥175	3	0.842	2.75 (0.73, 10.45)	87.7%	0.000

Supplementary Table 4 (continued)

		＜175	4		1.75 (0.70, 4.40)	71.9%	0.029
antibiotics	overall		5	0.000	2.46(0.98,6.18)	89.1%	0.000
	country	China	4	-	1.44 (0.82, 2.55)	71.4%,	0.015
		Abroad	1		36.92 (10.54, 129.38)	.%	-
	Study type	cohort study	3	0.000	1.08 (0.77, 1.51)	0%	0.648
		case-control study	2		10.41 (0.98, 110.77)	91.2%	0.001
	Sample size	≥175	1	0.001	3.33 (1.76, 6.28)	.%	-
		＜175	4		2.32 (0.75, 7.14)	90.3%	0.000
corticosteroid	overall		6	0.751	1.64(1.07, 2.53)	65%	0.000
	country	China	4	0.145	1.33(0.98, 1.80)	33.2%	0.213
		Abroad	2		4.94(2.27, 10.73)	0%	0.853
	Study type	cohort study	4	0.036	1.16(0.86, 1.57)	0%	0.530
		case-control study	2		2.84(1.05, 7.69)	79.3%	0.028
	Sample size	≥175	4	0.303	1.32(0.69, 2.53)	77.5%	0.035
		＜175	2		2.04(1.00, 4.16)	66.7%	0.029

Note: CLD: chronic liver disease; ALT: alanine aminotransferase; AST: aspartate aminotransferase; GGT: gamma-glutamyl transferase; TBIL: total bilirubin; ALB: albumin; PT: prothrombin time; APTT: activated partial thromboplastin time; INR: International Normalized Ratio.

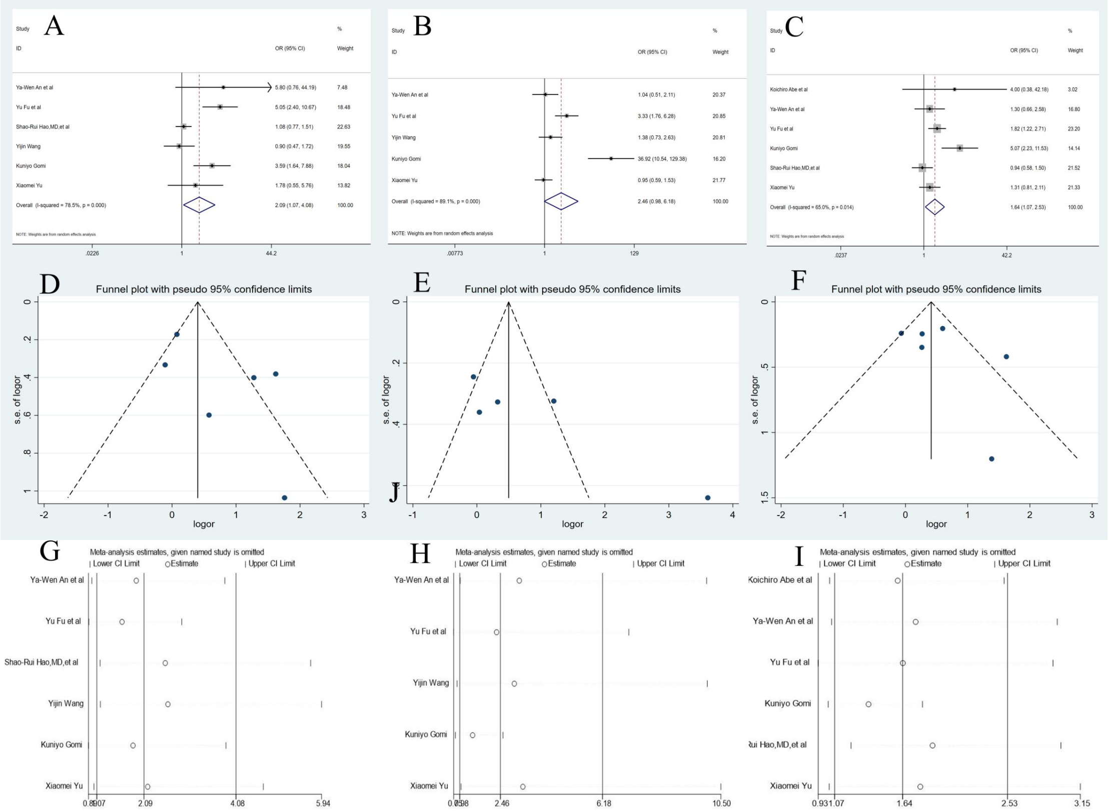

Supplementary Figure 1 The relationship between drugs and liver injury. (A-C) Forest plots of the included studies evaluating abnormal liver chemistries in patients receiving lopinavir or ritonavir, antibiotics, and corticosteroid treatments, respectively.(D-F) Funnel plot of the included studies evaluating abnormal liver chemistries in patients receiving lopinavir or ritonavir, antibiotics, and corticosteroid treatments, respectively.(G-I) Sensitivity analysis of the serum liver chemistries in patients receiving lopinavir or ritonavir, antibiotics, and corticosteroid treatments, respectively.
